# jMorp: Japanese Multi-Omics Reference Panel update report 2023

**DOI:** 10.1093/nar/gkad978

**Published:** 2023-11-01

**Authors:** Shu Tadaka, Junko Kawashima, Eiji Hishinuma, Sakae Saito, Yasunobu Okamura, Akihito Otsuki, Kaname Kojima, Shohei Komaki, Yuichi Aoki, Takanari Kanno, Daisuke Saigusa, Jin Inoue, Matsuyuki Shirota, Jun Takayama, Fumiki Katsuoka, Atsushi Shimizu, Gen Tamiya, Ritsuko Shimizu, Masahiro Hiratsuka, Ikuko N Motoike, Seizo Koshiba, Makoto Sasaki, Masayuki Yamamoto, Kengo Kinoshita

**Affiliations:** Tohoku Medical Megabank Organization, Tohoku University, Sendai, Miyagi 980-8573, Japan; Tohoku Medical Megabank Organization, Tohoku University, Sendai, Miyagi 980-8573, Japan; Tohoku Medical Megabank Organization, Tohoku University, Sendai, Miyagi 980-8573, Japan; Advanced Research Center for Innovations in Next-Generation Medicine, Tohoku University, Sendai, Miyagi 980-8573, Japan; Tohoku Medical Megabank Organization, Tohoku University, Sendai, Miyagi 980-8573, Japan; Advanced Research Center for Innovations in Next-Generation Medicine, Tohoku University, Sendai, Miyagi 980-8573, Japan; Tohoku Medical Megabank Organization, Tohoku University, Sendai, Miyagi 980-8573, Japan; Advanced Research Center for Innovations in Next-Generation Medicine, Tohoku University, Sendai, Miyagi 980-8573, Japan; Tohoku Medical Megabank Organization, Tohoku University, Sendai, Miyagi 980-8573, Japan; Graduate School of Medicine, Tohoku University, Sendai, Miyagi 980-8575, Japan; Tohoku Medical Megabank Organization, Tohoku University, Sendai, Miyagi 980-8573, Japan; Iwate Tohoku Medical Megabank Organization, Iwate Medical University, Shiwa-gun, Iwate 028-3609, Japan; Tohoku Medical Megabank Organization, Tohoku University, Sendai, Miyagi 980-8573, Japan; Graduate School of Information Sciences, Tohoku University, Sendai, Miyagi 980-8579, Japan; Tohoku Medical Megabank Organization, Tohoku University, Sendai, Miyagi 980-8573, Japan; Tohoku Medical Megabank Organization, Tohoku University, Sendai, Miyagi 980-8573, Japan; Faculty of Pharma-Science, Teikyo University, Tokyo 173-8605, Japan; Tohoku Medical Megabank Organization, Tohoku University, Sendai, Miyagi 980-8573, Japan; Advanced Research Center for Innovations in Next-Generation Medicine, Tohoku University, Sendai, Miyagi 980-8573, Japan; Tohoku Medical Megabank Organization, Tohoku University, Sendai, Miyagi 980-8573, Japan; Graduate School of Medicine, Tohoku University, Sendai, Miyagi 980-8575, Japan; Tohoku Medical Megabank Organization, Tohoku University, Sendai, Miyagi 980-8573, Japan; Advanced Research Center for Innovations in Next-Generation Medicine, Tohoku University, Sendai, Miyagi 980-8573, Japan; Graduate School of Medicine, Tohoku University, Sendai, Miyagi 980-8575, Japan; RIKEN Center for Advanced Intelligence Project, Tokyo 103-0027, Japan; Tohoku Medical Megabank Organization, Tohoku University, Sendai, Miyagi 980-8573, Japan; Advanced Research Center for Innovations in Next-Generation Medicine, Tohoku University, Sendai, Miyagi 980-8573, Japan; Tohoku Medical Megabank Organization, Tohoku University, Sendai, Miyagi 980-8573, Japan; Iwate Tohoku Medical Megabank Organization, Iwate Medical University, Shiwa-gun, Iwate 028-3609, Japan; Tohoku Medical Megabank Organization, Tohoku University, Sendai, Miyagi 980-8573, Japan; Advanced Research Center for Innovations in Next-Generation Medicine, Tohoku University, Sendai, Miyagi 980-8573, Japan; Graduate School of Medicine, Tohoku University, Sendai, Miyagi 980-8575, Japan; RIKEN Center for Advanced Intelligence Project, Tokyo 103-0027, Japan; Tohoku Medical Megabank Organization, Tohoku University, Sendai, Miyagi 980-8573, Japan; Advanced Research Center for Innovations in Next-Generation Medicine, Tohoku University, Sendai, Miyagi 980-8573, Japan; Graduate School of Medicine, Tohoku University, Sendai, Miyagi 980-8575, Japan; Tohoku Medical Megabank Organization, Tohoku University, Sendai, Miyagi 980-8573, Japan; Advanced Research Center for Innovations in Next-Generation Medicine, Tohoku University, Sendai, Miyagi 980-8573, Japan; Graduate School of Pharmaceutical Sciences, Tohoku University, Sendai, Miyagi 980-8578, Japan; Tohoku Medical Megabank Organization, Tohoku University, Sendai, Miyagi 980-8573, Japan; Graduate School of Information Sciences, Tohoku University, Sendai, Miyagi 980-8579, Japan; Tohoku Medical Megabank Organization, Tohoku University, Sendai, Miyagi 980-8573, Japan; Advanced Research Center for Innovations in Next-Generation Medicine, Tohoku University, Sendai, Miyagi 980-8573, Japan; Iwate Tohoku Medical Megabank Organization, Iwate Medical University, Shiwa-gun, Iwate 028-3609, Japan; Tohoku Medical Megabank Organization, Tohoku University, Sendai, Miyagi 980-8573, Japan; Advanced Research Center for Innovations in Next-Generation Medicine, Tohoku University, Sendai, Miyagi 980-8573, Japan; Tohoku Medical Megabank Organization, Tohoku University, Sendai, Miyagi 980-8573, Japan; Advanced Research Center for Innovations in Next-Generation Medicine, Tohoku University, Sendai, Miyagi 980-8573, Japan; Graduate School of Information Sciences, Tohoku University, Sendai, Miyagi 980-8579, Japan

## Abstract

Modern medicine is increasingly focused on personalized medicine, and multi-omics data is crucial in understanding biological phenomena and disease mechanisms. Each ethnic group has its unique genetic background with specific genomic variations influencing disease risk and drug response. Therefore, multi-omics data from specific ethnic populations are essential for the effective implementation of personalized medicine. Various prospective cohort studies, such as the UK Biobank, All of Us and Lifelines, have been conducted worldwide. The Tohoku Medical Megabank project was initiated after the Great East Japan Earthquake in 2011. It collects biological specimens and conducts genome and omics analyses to build a basis for personalized medicine. Summary statistical data from these analyses are available in the jMorp web database (https://jmorp.megabank.tohoku.ac.jp), which provides a multidimensional approach to the diversity of the Japanese population. jMorp was launched in 2015 as a public database for plasma metabolome and proteome analyses and has been continuously updated. The current update will significantly expand the scale of the data (metabolome, genome, transcriptome, and metagenome). In addition, the user interface and backend server implementations were rewritten to improve the connectivity between the items stored in jMorp. This paper provides an overview of the new version of the jMorp.

## Graphical Abstract

**Graphical Abstract:**
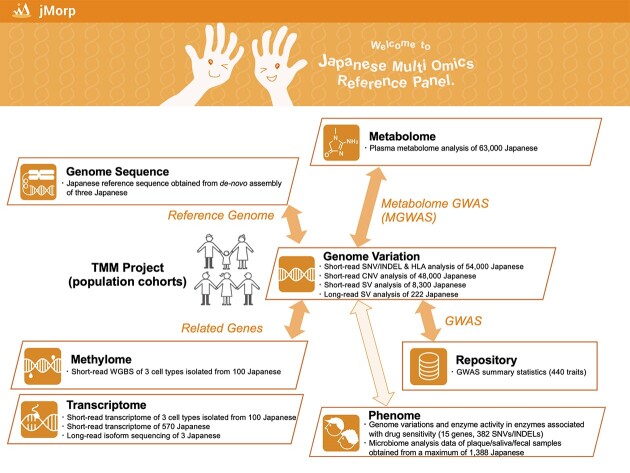

## Introduction

The field of personalized medicine is rapidly advancing with the increasing availability of multi-omics data which provide a deeper understanding of individual biological characteristics and disease mechanisms. One area of interest is genome-based drug discovery, which can contribute to personalized medicine. Additionally, it is important to note that different ethnic groups have unique genetic backgrounds and diversity in their genomes, which affect their susceptibility to certain diseases and their response to various medications. Therefore, to apply personalized medicine to a particular ethnic population, multi-omics data are indispensable.

Prospective cohort studies have been conducted around the world, such as the UK Biobank (1) in the United Kingdom, All of Us (2) in the United States, and LifeLines (3,4) in the Netherlands, to deliver personalized medicine to each population. As cohort studies progress, web databases displaying the results of analyses of the data collected, such as gnomAD (5) (https://gnomad.broadinstitute.org/) and deCAF (6) (https://decaf.decode.com/), have been developed. The Tohoku Medical Megabank (TMM) project (7) was initiated after the Great East Japan Earthquake in 2011. The project is conducting two prospective cohort studies of Japanese people to realize constructive regeneration and recovery from the great disaster: one is a community-based cohort (TMM CommCohort Study) (8), and the other is a Birth and Three-Generation cohort (TMM BirThree Cohort) (9). The TMM Project has approximately 150,000 participants involved in the two cohort studies which are used to collect biological specimens and perform typical genomic and omics analyses. Statistics from these analyses are widely available through the Japanese Multi-Omics Reference Panel (jMorp; https://jmorp.megabank.tohoku.ac.jp) web database. jMorp uses a multidimensional, multidisciplinary approach to handle the diversity of the Japanese population from multiple perspectives. It serves as a source of information for promoting personalized medicine, especially through identifying mutations related to cancer and undiagnosed rare diseases.

jMorp was launched in 2015 as a public plasma metabolome and proteome analysis database and is updated annually. In the 2020 update, new types of data, including genome sequence (reference genomes) (10), genome variation (SNV and short-INDEL) information (11), genome methylation information (12), transcriptome information, and a GWAS summary statistics repository, were added to jMorp, as reported in a previous paper (13). In this update, we describe the extensive enhancement and addition of new types of data, such as structural and copy number variations regarding the genomics, metagenomics, and pharmacogenomics (PGx) of the Japanese population. In addition, the user interface and backend server implementations were comprehensively rewritten to enhance the links between the data stored in jMorp. This paper provides an overview of the data in the latest jMorp database and its use.

## Materials and methods

### Genome variation analysis

#### SNV and short-INDEL analysis

The 54KJPN panel was derived from the whole genome sequencing of 69,014 Japanese individuals. The complete methodology has been described elsewhere. Genomic DNA was obtained from peripheral blood, saliva, or cord blood samples. It was sequenced on several platforms, including the Illumina HiSeq 2500, HiSeq X Five, NovaSeq 6000, MGI DNBSeq G400, and DNBSeq T7. Notably, Toshiba undertook sequencing on a HiSeq X Five, and parts of the NovaSeq sequencing were managed by TaKaRa Bio Co. Ltd., iLac Inc. and HaploPharma Inc.

The resequencing process adhered to GATK Best Practices. Reads from each sample in FASTQ format were aligned to the GRCh38 human reference sequence, primarily using either BWA (14) 0.7.15 or BWA-mem2 2.1. Following this alignment, SNV/INDEL calling was performed using GATK HaplotypeCaller (15). Before initiating variant calls, the alignments underwent base-quality score recalibration (BQSR) using the GATK BaseRecalibrator tool. After individual variant calling for all samples, multisample joint genotyping was performed using Sentieon Genomics tools. These variants were annotated using the GATK VariantQualityScoreRecalibration (VQSR) tool.

The allele frequency for each variant was calculated from the genotype information obtained from the joint genotyping analysis. Since related samples were present in this genotyping analysis, a filtering process was necessary to isolate unrelated samples before determining the allele frequencies. This filtration was achieved using KING 2.3.1 software (16). From the entire set, 54,302 samples were selected by KING, culminating in forming the 54KJPN allele frequency panel. A separate article provides a more in-depth exploration of the construction of the 54 KJPN panel. A summary of the samples in the 54KJPN panel is provided in Supplementary Table S1.

#### Variation IDs of SNVs ans INDELs to link from external databases

The current version of jMorp assigns a unique Variation ID to each SNV and INDEL using VariantKey (17), with a digit to specify the reference genome, which is used as a part of the URL. For example, rs671 at chr12:111803962 G/A on GRCh38 has a unique ID of 06354ff1d08c00000, and the URL of this variant is https://jmorp.megabank.tohoku.ac.jp/genome-variations/sr-snvindel/06354ff1d08c00000. The first character of the variation ID is the reference genome. (0: GRCh38, and 1: GRCh37), and the remaining characters were calculated using VariantKey. Using VariantKey, it is possible to express the genome coordinates and alleles of an SNV/INDEL in a short form, which enables users to link all variation pages in jMorp with this URL.

#### HLA analysis

HLA analysis and allele frequency calculations were performed on the same population as the 54KJPN panel. A summary of the samples in the 54KJPN panel is provided in Supplementary Table S1. Genotypes in the G group were obtained for the following HLA genes with HLA*LA (18) 1.0.3 from the short-read sequencing data for samples in the 54KJPN: HLA-A, -B, -C, -E, -F, -G, -DRB1, -DRB3, -DRB4, -DQA1, -DQB1, -DPA1 and -DPB1. The obtained genotypes were reclassified into the P group based on information from the IPD-IMGT/HLA HLA Database (19), Release 3.53 (2023–07) Version.

#### CNV analysis

We used GermlineCNVCaller from GATK for our CNV analysis based on our pipeline on GATK. Due to the challenge of processing hundreds of samples simultaneously, we broke down the process as follows: First, we ran a Germline Cohort Workflow, analyzing 200 samples grouped by sequencer and sequencing agency. Each of the 200 samples was subjected to a separate Germline Case Workflow. We filtered out samples with unusual amplification and loss figures using the interquartile range (IQR) method on the 54KJPN datasets to ensure that our results were reliable. Through this process, we determined the frequencies of 48,874 samples. A summary of the CNV panel samples is presented in Supplementary Table S2.

### Transcriptome analysis

A separate paper will describe the whole-blood transcriptome analysis, but a brief overview follows. In the TMM project whole blood samples were collected from 4,337 participants in PAXgene(R) Blood RNA Tubes and stored at −80°C. To observe age-related differences in expression levels, 576 samples from males and females in their 30s and 60s were selected from among the 4,337 samples, and transcriptome analysis was performed. Supplementary Table S3 summarizes samples from the whole-blood transcriptome dataset. Total RNA was extracted using the PAXgene(R) Blood RNA Kit. Ribosomal RNA was removed, and RNA libraries were prepared. DNA libraries were constructed using MGIEasy RNA Directional Library Prep Sets through the RNA libraries. The DNA libraries were sequenced using an MGI DNBSEQ-G400 sequencer. Raw sequence reads were quality-controlled for data analysis using Trimmomatic software (20). High-quality reads were aligned to the GRCh38 human genome sequence using STAR (21), and read counts and expression levels were calculated using RSEM (22). The expression levels of the individual genes were adjusted by removing the effects of globin using an in-house Python script.

### Metabolome analysis

We have referred to our previous report (13) and other metabolome analyses (23–26). Briefly, NMR metabolome analysis was performed on plasma samples stored at -80°C. The metabolites were extracted and subjected to NMR experiments at 298 K using Bruker 600 MHz spectrometers. We utilized standard NOESY and CPMG spectra for each sample and analyzed the data using the Chenomx NMR Suite. Our in-house software facilitates automatic metabolite quantification (Aoki *et al.*, in preparation). See Supplementary Table S4 for the NMR-analyzed samples.

For the MS metabolome, we employed G-Met analysis by HPLC-Q-FT/MS and WT-Met analysis by GC-MS/MS on cohort plasma samples following previously established protocols (24,27–29). Metabolite area ratios adjusted for batch variation using gQC analyses (29) were derived from the MS results. For UHPLC-MS/MS-based WT-Met analysis, cohort plasma samples were prepared using the AbsoluteIDQ(R) p180 and MxP(R) Quant 500 kits. The LC and FIA modes for UHPLC-MS/MS and the related parameters were set according to the kit guidelines. Final values were computed and standardized using the MetIDQ Oxygen software. Supplementary Table S4 shows the counts of the MS-analyzed samples.

### Metagenome analysis

For the 16S-v4 region analysis and the 16S-v3/v4 region analysis, details are described in Saito *et al.* (30). Briefly, amplicon sequencing analysis was performed using the Illumina MiSeq Platform on saliva and plaque samples from the TMM cohort participants. The reads obtained by sequencing were analyzed using QIIME2 (31), and the relative abundance of microbes in each sample was obtained. In total, 1,289 and 1,388 samples were subjected to 16S-v4 analysis and 16S-v3/v4 analysis, respectively.

Details of shotgun metagenomic analysis will be described elsewhere. The summary of the analysis method is as follows: metagenome sequencing analysis was performed using the Illumina NovaSeq 6000 on 315 fecal samples from the TMM cohorts. The raw sequence reads were quality-controlled using fastp (32). Subsequently, host-derived reads were identified and removed using BMTagger (33) with the GRCh38 human genome sequence. The relative abundance of microbes in each sample was estimated from the cleaned reads using MetaPhlAn3 (34).

### Enzymatic activity of CYP genes

Changes in the enzymatic activities of CYP variants have been previously described (35–39). First, a resequencing analysis was performed using the Sanger method to evaluate the accuracy of the genetic polymorphism information identified by NGS. Next, we constructed expression vectors inserted CYP wild-type and variant cDNAs. All constructs were confirmed by Sanger sequencing. The expression vector used was pcDNA3.4, a mammalian expression vector (Thermo Fisher Scientific, Waltham, MA, USA). Cytochrome P450 oxidoreductase (CPR) and cytochrome b5 cDNA insertion vectors have also been developed. CYPs were expressed in the human fetal kidney-derived 293FT cell line or the African green monkey kidney-derived COS-7 cell line with either wild-type or variant expression. For Western blotting, CYP protein expression was quantified using the Wes system and the Compass software for SW ver. 4.1.0 (ProteinSimple, San Jose, CA, USA). According to previous reports, CYP, CPR, and cytochrome b5 content were then measured (36). CYP activity of the wild-type and variants was assessed by enzyme reaction kinetic analysis using specific substrates for each CYP. For example, for CYP3A4, midazolam 1′-hydroxylation, and testosterone 6β-hydroxylation activities, and CYP2C9, S-warfarin 7-hydroxylation and tolbutamide 4-hydroxylation kinetic parameters (Km, Vmax and CLint) were calculated. Functional change analysis of drug-metabolizing enzyme variants, other than CYP genes, such as DPYD and DPYS, was performed in the same manner as that for CYPs (40,41). Genomic variants on the CYP genes were extracted from our genome variation panel, and thus, the variants can be found in the Japanese population. In that sense, our PGx data is population-specific data. Still, it is highly possible that the activity of CYPs would be similarly changed if the same variants were observed in other populations.

### jMorp web server implementation

In jMorp, most of the data reside in the PostgreSQL database (https://www.postgresql.org/). Each dataset has a dedicated PostgreSQL instance, which is unified into a single virtual instance using the postgres_fwd module. To manage web browser requests, the Hasura GraphQL engine (https://hasura.io/) is implemented ahead of the PostgreSQL server.

Here's how it works: Hasura, acting as a middleware, takes in GraphQL queries. It then translates these into SQL, directs them to the PostgreSQL servers, and returns the results in JSON format. This mechanism differs from the traditional REST API. GraphQL is prominent because it can tap multiple resources in a single query. In addition, it visualizes the relationships between resources as a graph, streamlining the search process for interconnected entries.

Supplementary Figure S1 shows a sample GraphQL query that provides a clearer picture. This query fetches data on the metabolite named ‘Glycine’ and any related GWAS analyses. The main ‘metabolite { …}’ structure filters by the metabolite's name. Meanwhile, nested blocks like ‘gwasTopHitSummary { …}’ and ‘variationSummary { …}’ source the top genome variations connected to that metabolite from the GWAS statistics. This exemplifies GraphQL’s capability to interact with multiple tables through one cohesive query, a feature that significantly complements databases such as jMorp, which consolidates diverse datasets.

### Genome browser plugin-implementation

JBrowse2 (42) was employed to deliver a consolidated view of diverse jMorp data, encompassing the SNV/INDEL reference genomes of 54KJPN, structural variations such as JSV1 and Japonica Array marker positions, and Manhattan plots of GWAS. In addition, non-jMorp data were analyzed using ClinVar, gnomAD, refSeq GENCODE, repeat masker, and dbSNP annotations (Supplementary Figure S2). JBrowse2 genome browser was used to analyze each reference genome. The current version of jMorp is equipped with three genome browsers: GRCh37, GRCh38 and JG2.1.0. To facilitate a more seamless integration with jMorp, we developed a plug-in that allows users to navigate directly to relevant pages. This plug-in also implements a function to search for and display data by calling the GraphQL API. While normal JBrowse only allows searches by gene name, jMorp's JBrowse also allows searches by dbSNP rs number and HGVS sequence variant nomenclature, such as dbSNP ‘ALDH2 p.Glu504Lys’. This has dramatically improved user experience.

## Results and discussions

### Overview and page structure of the jMorp

**Figure 1.**
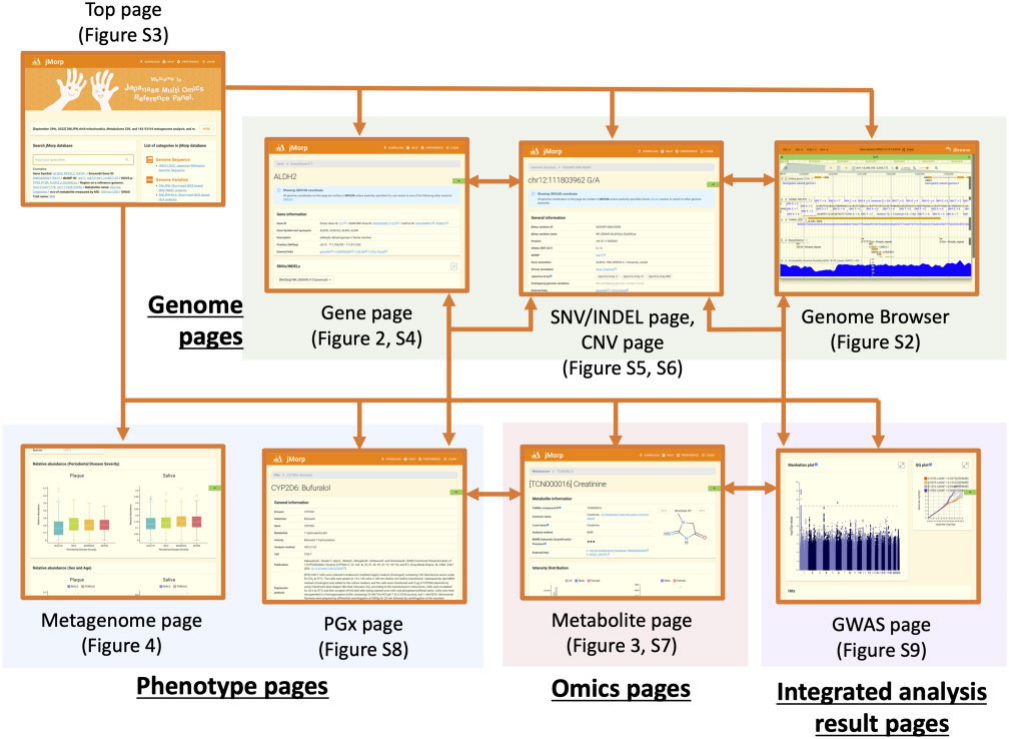
Page structure of the jMorp database.

Figure 1 shows the configuration of the main pages of jMorp, and Supplementary Table S5 provides a comprehensive list of the datasets included in jMorp along with those related to each page and differences from our previous report (13). The pages were grouped according to the data category displayed on the page, and the background colors represented the groups.

**Figure 2.**
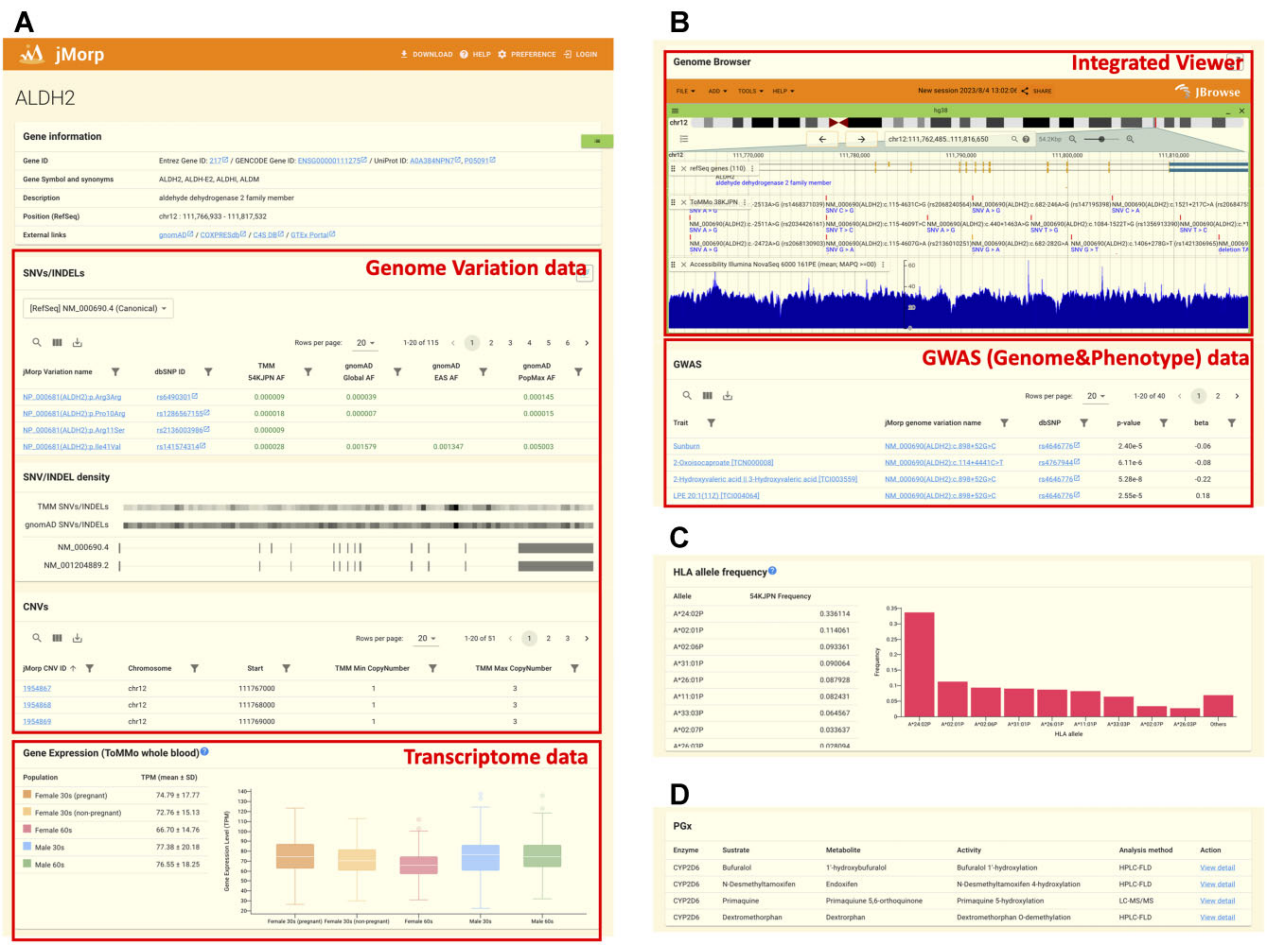
An example of a gene page. (**A**) Top of gene page for ALDH2 gene. The gene page aggregates and displays multiple layers of data, such as genome variation and transcriptome, and this helps users get a quick look at the diversity in the Japanese population from various perspectives. (**B**) Bottom of gene page for ALDH2 gene (continuation of (A)). (**C**) HLA allele frequency panel in gene page for HLA-A gene. (**D**) PGx link panel in gene page for CYP2D6 gene.

The Genome Pages consist of three types: The first is a gene-centric page, called the Gene page (Figure 2). It shows four types of data for each gene, including genome variations (SNVs/INDELs and CNV frequencies based on short-read WGS analysis of 54,000 and 48,000 participants from TMM cohorts, respectively (TMM whole genome panels)), methylation states determined by 300 whole bisulfite sequences, gene expression levels from short-read analysis of approximately 600 samples in TMM cohorts, long-read analysis of three Japanese males, and GWAS analysis results. (See the next section for further details.) The second type is variant-oriented and includes the SNV/INDEL (Supplementary Figure S5) and CNV pages (Supplementary Figure S6). The SNV/INDEL page was generated for each TMM whole-genome panel variant. It presents some details on SNVs and INDELs, such as allele frequency from the whole genome panel and the gnomAD (5) database, gene annotations, correlation with other SNVs and INDELs calculated based on TMM WGS panels, and liftover results for other genome assemblies. The CNV page shows a table and a plot of the number of samples per copy number variation. The third type is the Genome Browser page (Supplementary Figure S2) which can be used to visualize genome-related data, including genome sequences, genome variation (SNV/INDEL, CNV, SV) information, genetic maps (or linkage disequilibrium maps), marker sites of Japanese-specific SNV arrays (Japonica Array (43,44)), genome accessibility data, GWAS results, and publicly available datasets such as dbSNP (45) and ClinVar (46).

**Figure 3.**
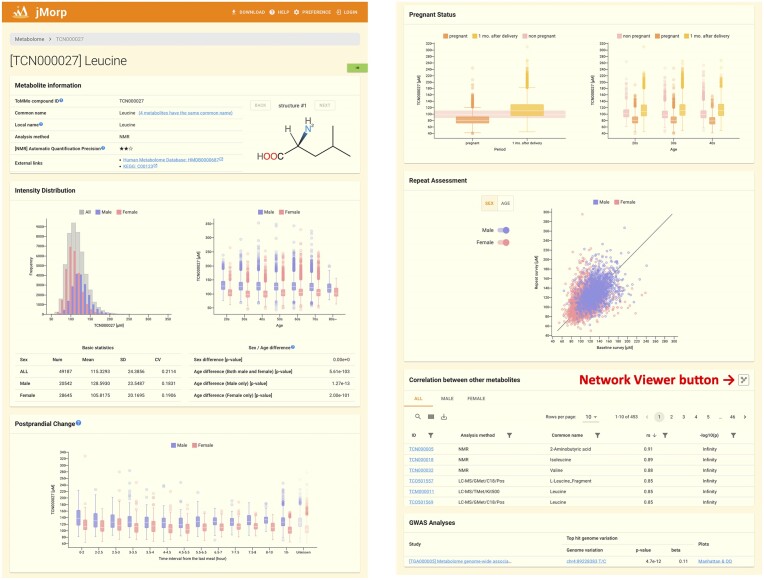
Metabolite page for leucine (TCN000027).

**Figure 4.**
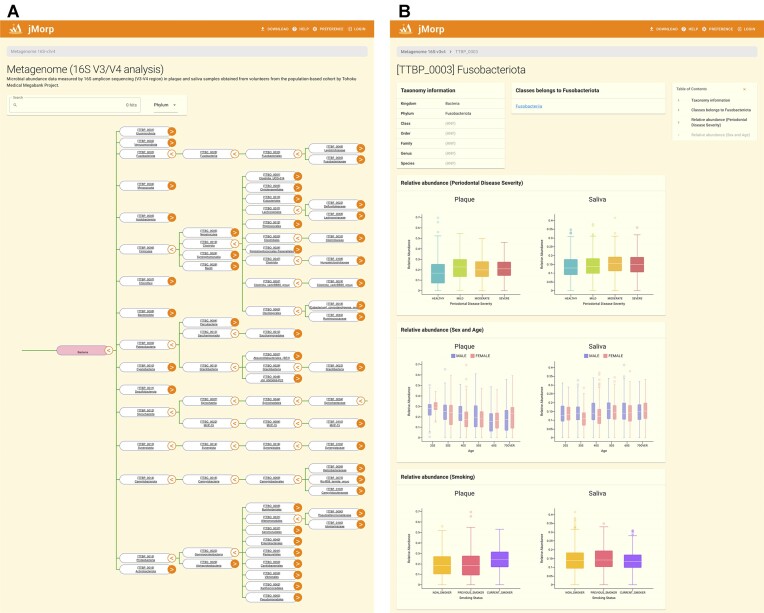
Metagenome page examples. (**A**) Microbe tree page for 16S-v3/v4 dataset. The phylogenetic tree of the microbe is displayed on this page. Users can view lower-level microbes by clicking the button on the right end of the node. In addition, by clicking on the name of a microbe, users are navigated to an analysis result page (Figure 4B). (**B**) Metagenome analysis result page for Fusobacteriota (TTBP_0003).

The Omics pages now contain Metabolite pages (Figure 3), including the frequency distribution of metabolite concentrations, trends by age and sex, and correlations with other metabolites obtained by plasma metabolome analyses of 63,000 participants from the TMM cohorts. The Phenotype pages consist of a metagenome page and a PGx page. The metagenome page (Figure 4) displays microbial abundance data obtained by 16S amplicon sequencing of plaque and saliva samples and shotgun sequencing of fecal samples obtained from participants in the TMM cohorts. The PGx page (Supplementary Figure S8) displays the results of *in vitro* analysis of enzyme activity changes for 14 drug-metabolizing enzymes, with genome variations involving 382 amino acid substitutions associated with drug susceptibility.

The Integrated analysis pages consist of GWAS pages (Supplementary Figure S9), each providing summary statistics of the GWAS performed as part of the TMM project. These pages are interconnected with other pages, as indicated by the arrows in Figure 1 so that users can quickly access multiple types of data in an integrated manner. For example, the Metabolite page (Figure 3) contains a panel that lists the results of GWAS performed on that metabolite as a trait, and the panel is linked to the GWAS page. The GWAS page (Supplementary Figure S9) displays a list of genomic variations with significant p-values in the GWAS analysis, and users can use the list to jump to the SNV/INDEL and gene pages.

The jMorp database is organized into multiple data layers that are easily navigated, enabling a comprehensive understanding of the diverse Japanese population. In later sections of this paper, we focus on the latest updates and developments made to the gene, metabolite, metagenome, and GWAS pages. Supplementary Figures S2−S9 provide screenshots and summaries of the remaining pages.

### Gene page

The best example of the multilayered nature of jMorp is the gene page. Searching for the gene name as a keyword in the search box at the top of the start page opens the corresponding gene page. For example, Figure 2A and B shows the jMorp gene page for ALDH2. The gene information panel at the top of the page shows the gene's ID, symbol, and position in a reference genome, as well as links to external databases. Below the information panel, the analysis results related to the gene from the TMM project are displayed from genomic to phenotypic information in the order of the central dogma.

Genomic variation data are displayed in three panels: SNVs/INDELs, SNV/INDEL density and CNVs. The SNVs/INDELs panel shows the locations and allele frequencies of SNVs and INDELs. In addition, we provide allele frequency information from our 54KJPN panel, which is based on the whole genome data of approximately 54,000 Japanese individuals, as well as from the gnomAD (5) database operated by the Broad Institute. By displaying the allele frequencies of 54KJPN and gnomAD side-by-side, users can easily confirm the differences between the Japanese and other ethnic groups. Clicking on the SNV/INDEL ID directs the user to the SNV/INDEL detailed page, which describes the content in the next section.

The SNV/INDEL density panel graphically displays the density of SNVs and short INDELs in the gene. As shown in Figure 2A, the panel contains four horizontal bars. The first and second bars show the density of the genome variations calculated using the 54KJPN panel and gnomAD, respectively, each representing the length of the gene. SNV and INDEL densities were calculated for each 10 kb bin and are shown with different color intensities, where black indicates a high number density of variants in the bin and white indicates lower densities. The SNV/INDEL density panel graphically displays the densities of SNVs and short INDELs in a gene. The two horizontal bars display the locations of exons in the gene for the corresponding transcripts. Horizontal bars indicate whether three or more transcripts are reported for a gene. Mutations are unlikely to occur in regions that are not important for maintaining gene function. Thus, this density panel can be used to determine where mutations are likely to be introduced and how they differ in each population.

The CNVs panel lists the copy number variations (CNVs) of a gene. CNV frequency data were based on short-read WGS analyses of approximately 48,000 participants in the TMM cohorts (JCNVv1 panel). The panel displays a table containing the ID assigned to each CNV, its position in the reference genome, and minimum and maximum copy numbers. Detailed information is obtained by clicking on the CNV ID (Supplementary Figure S6).

Transcriptome data for genome variation-related data are available below. jMorp now provides three kinds of transcriptome data: (i) gene expression of whole blood samples; (ii) gene expression of CD4 T-lymphocytes, monocytes, and neutrophils and (iii) full-length transcriptome using ISO-seq technology (47). In this update, (i) and (iii) have been added. (a) RNA-seq of whole blood samples was performed to provide a reference distribution of the transcriptome in a Japanese population. Since gene expression level is sensitive to sex and age, we included samples of females and males in their 30s and 60s. In addition, some of the younger females were pregnant; therefore, we furtheir divided them into pregnant and non-pregnant groups and showed the distribution of each category. For the full-length transcriptome (iii), we provided a reference structure for the transcript using long-read sequencing technology. The details of the full-length transcriptome have been previously described by Otsuki *et al.* (47). Note that (i) and (iii) target specific cells for gene expression analysis. Therefore, there is a limitation that genes specifically expressed in those cells can be captured. In addition, it should be noted that (i) currently targets only genes on autosomes and does not include gene expression information on sex chromosomes. Each (i), (ii) and (iii) has both advantages and disadvantages. Whole blood data (ii) is considered helpful in the sense that it includes gene expressions that cover a variety of cells. On the other hand, we believe that (i) and (iii) are also valuable because they evaluate transcripts of specific cells with high precision. In this way, jMorp shows gene expression data at various resolutions, making it possible to observe the diversity of Japanese people from multiple perspectives.

The genome browser (Figure 2B) is displayed below the transcriptome analysis data panel. The genome browser can graphically check gene structure, SNVs/INDELs, CNV and other genome-related information.

The GWAS results are also available below the genome browser. jMorp includes a repository for aggregating GWAS performed in the TMM project that contains GWAS study summaries and summary statistics data. The GWAS panel extracts and displays GWAS analyses in which a significant variant is found in the gene from the GWAS summary statistics file in jMorp. Users can check the trait overview by clicking on the name of the GWAS trait. If a GWAS trait, such as a metabolite, is an item recorded in jMorp, it can jump to that entry by following a link.

In addition, the gene page also includes a panel that displays genome methylation information (Supplementary Figure Supplementary Figure S4A) and transcriptome information (Supplementary Figure S4B) recorded in the iMETHYL (12) database, which is operated by a partner of TMM, Iwate Medical Megabank (IMM), and a panel that displays transcriptome (ISO-Seq) data from long-read WGS analysis (Supplementary Figure S4C), which were omitted from Figure 2 due to the limit of figure size.

We have provided additional information on the HLA-and drug metabolism-related genes. Figure 2C demonstrates the HLA allele frequency panel displayed on the HLA-A page. The gene encoding HLA is located on the short arm of chromosome 6 and has multiple alleles. In addition to many alleles, there are pseudogenes with similar sequence structures; therefore, accurate HLA typing is difficult with short-read WGS-based SNV/INDEL analysis methods. The SNV/INDEL panel also displayed the SNV/INDEL frequency information for the HLA gene. However, the allele frequencies displayed in the panel are based on ordinal germline variant analysis using normal short-read WGS, and the results may require corrections. Therefore, in jMorp, the results analyzed using the HLA-dedicated variant caller are displayed on the HLA panel. Similar to the HLA genes, the CYP2D6 gene is shown in the PGx panel on the gene page (Figure 2D) as an example of drug susceptivity-related genes, where jMorp provides enzymatic activity measurement data for set of CYP450 genes. The link of ‘view detail’ in the PGx table on the gene page directs the user to the information on the enzymatic activity of the enzyme for a representative drug molecule related to CYP450 on the PGx page (Supplementary Figure S8).

### Metabolome page

jMorp contains NMR and MS metabolome analysis data for the plasma of approximately 63,000 TMM cohort participants. A list of metabolomic analyses and the number of samples analyzed are provided in Supplementary Table S4. The list of metabolites can also be viewed by clicking the ‘Metabolome 2023’ link on the right side of the jMorp top page (Supplementary Figure S3). The current version of jMorp contains 1,331 metabolites. By clicking the name of the metabolite of interest from the list of metabolites, it is possible to move to a page that displays the details of the metabolite. Figure 3 shows the metabolome page of leucine data measured by NMR as an example.

At the top of the page is a Metabolite Information panel that displays basic information such as the ID assigned, metabolite name, metabolite structural formula, measurement method, and links to external databases. The Intensity Distribution panel is presented below. This panel presents a histogram showing the concentration distribution of each metabolite in the TMM cohort participants. The concentrations were further grouped by age and sex and are represented in boxplots. Accompanying the visual representations is a table of summary statistics, which includes the number of samples analyzed, mean concentration, SD, and CV. Additionally, statistical test results regarding differences in sex and age are provided.

The following Postprandial Change panel shows the changes in metabolite concentrations after meals. The concentration of some metabolites changes with food consumption, and this panel can be used to identify differences in plasma metabolite concentrations depending on the elapsed time after a meal.

Pregnancy status is another significant factor in changing the metabolome state; therefore, boxplots in the pregnancy status panel illustrate metabolite concentration differences between pregnant, one-month post-delivery, and non-pregnant females. The second plot further breaks down these groups by age (i.e. 20s, 30s and 40s).

The metabolome state is dynamic; therefore, the concentrations change with each assessment. The Repeat Assessment panel features a scatter plot comparing metabolite concentrations from the baseline and 3–5-year follow-up surveys of the TMM cohort. Each dot, colored according to sex and age, represents a sample showing changes in concentration over time.

A correlation of metabolite concentrations among the dataset helps understand the changes in metabolites. Therefore, a correlation table of metabolites is provided below the ‘Repeat Assessment’ panel. This table lists Spearman's rank coefficients (|*r*s| > 0.2) and *P*-values. These relationships can also be examined from a Network View using the ‘Network View’ button (Figure 3 and Supplementary Figure S7). At the end of the metabolite page, the results of the GWAS analysis with metabolite concentration as a trait are displayed if any significant associations were found in the TMM cohort. It is possible to jump to the gene and SNV/INDEL pages using this GWAS Analysis panel, making it possible to examine metabolites from both omics and genome perspectives. This interconnection among different types of omics data is the most important feature of jMorp.

Additional detailed statistics (mean, standard deviation, and median values for each 5-year age group and sex) of the metabolites measured by NMR, designated as the metabolic index, is also available (the panel is omitted from Figure 3 due to the limited size of the figure).

### Metagenome page

The latest version of jMorp provides access to three metagenomic datasets derived from TMM cohorts. The first dataset comprises microbial abundance data from both plaque and saliva samples collected from 1,200 volunteers. These data were determined using 16S amplicon sequencing targeting the V4 region. The second dataset presented microbial abundance data from plaque and saliva samples from another set of 1,300 volunteers. This set was assessed using 16S amplicon sequencing, focusing on both the V3 and V4 regions. Finally, the third dataset features microbial abundance data from fecal samples of 300 volunteers, measured using shotgun metagenome sequencing. Access to these datasets is conveniently available via the links on the top page. A phylogenetic tree representing the microbes within the chosen dataset appears upon the selection of any of these links, as illustrated in Figure 4A. Furthermore, users can conduct a more detailed microbial analysis by clicking on any tree node, as shown in Figure 4B. Notably, the nature of the data displayed on the metagenome page differed depending on the analysis method applied. For datasets obtained via 16S-v4 and 16S-v3/v4 sequencing, this page highlights the relative microbial abundance across various groups. These groups were categorized based on the sample type (e.g. dental plaque or tongue debris), severity of periodontal disease, sex, age, smoking history and respiratory function. On the other hand, the data from the shotgun sequencing dataset is categorized to show relative microbial abundance based on gender, age, and fecal condition, with the latter being determined using the Bristol Scale.

### GWAS summary statistics repository

The jMorp GWAS summary statistics repository stores GWAS analyses organized by study and publications usually describe each study. Currently, 11 GWAS have been registered, and GWAS summary statistics files for 401 traits are stored in jMorp. To the best of our knowledge, this is the largest repository of GWAS analyses of the Japanese population.

The jMorp GWAS repository has two main pages: the GWAS study list (Supplementary Figure S9A), and the GWAS trait list. Users can directly jump to these pages from the top page of jMorp, but they usually access each GWAS result from the gene and metabolome pages. The GWAS study list shows all studies included in jMorp, whereas the GWAS trait list provides all available traits. By clicking on the GWAS study title on the table, users can access the GWAS study detail page (Supplementary Figure S9B), which includes the study title, abstract, and analysis method summary (sample type, number of samples, and analysis software). Each GWAS has unique methods detailed on its page, including an overview and relevant publication links. This page lists the analyzed sample, platform, reference genome, software details, and variant filtering criteria. Some studies uploaded data to jMorp's GWAS repository before publication, rendering publication links unavailable. The uploaded summary statistics were processed using PheWeb (48) to extract significant genomic variations. These variations were then integrated into the jMorp database and displayed on the GWAS analysis detail (Supplementary Figure S9C), Gene page and Metabolite page.

### Future directions

This paper outlines the current contents of the jMorp database and its use. We have been continuously developing and operating jMorp since 2015 and will continue to expand jMorp. In future extensions, we will focus on two main points: (i) expansion of the number of samples to be analyzed and (ii) enhancement of information linking multiple layers. By increasing the number of samples and improving the analysis methods of each dataset, we aim to increase the accuracy of analysis results. In addition, by expanding the links between each layer and the links between the hierarchies, we would like to contribute as a useful resource when examining complex biological phenomena.

## Supplementary Material

gkad978_supplemental_file

## Data Availability

The jMorp database is freely available at https://jmorp.megabank.tohoku.ac.jp. We clarified the Conditions of Use at https://jmorp.megabank.tohoku.ac.jp/help/conditions-of-use.
