# Supplementary material for: jMorp: Japanese Multi-Omics Reference Panel update report 2023
Source: Nucleic Acids Res. 2023 Nov 1;52(D1):D622–32. doi: 10.1093/nar/gkad978 (PMC10767895; doi:10.1093/nar/gkad978)
Supplement: gkad978_supplemental_file [file gkad978_supplemental_file.pdf]

jMorp: Japanese Multi-Omics Reference Panel update report 2023

Supplementary Materials

Shu Tadaka[1,+], Junko Kawashima[1,+], Eiji Hishinuma[1,2,+], Sakae Saito[1,2], Yasunobu Okamura[1,2], Akihito Otsuki[1,3], Kaname Kojima[1], Shohei Komaki[4], Yuichi Aoki[1,5], Takanari Kanno[1], Daisuke Saigusa[1,6], Jin Inoue[1,2], Matsuyuki Shirota[1,3], Jun Takayama[1,2,3,7], Fumiki Katsuoka[1,2], Atsushi Shimizu[1,4], Gen Tamiya[1,2,3,7], Ritsuko Shimizu[1,2,3], Masahiro Hiratsuka[1,2,8], Ikuko N Motoike[1,5], Seizo Koshiba[1,2], Makoto Sasaki[4], Masayuki Yamamoto[1,2], Kengo Kinoshita[1,2,5,*]

1. Tohoku Medical Megabank Organization, Tohoku University, Sendai, Miyagi, 980-8573, Japan
2. Advanced Research Center for Innovations in Next-Generation Medicine, Tohoku University, Sendai, Miyagi, 980-8573, Japan
3. Graduate School of Medicine, Tohoku University, Sendai, Miyagi, 980-8575, Japan
4. Iwate Tohoku Medical Megabank Organization, Iwate Medical University, Shiwa-gun, Iwate, 028-3609, Japan
5. Graduate School of Information Sciences, Tohoku University, Sendai, Miyagi, 980-8579, Japan
6. Faculty of Pharma-Science, Teikyo University, Tokyo, 173-8605, Japan.
7. RIKEN Center for Advanced Intelligence Project, Tokyo, 103-0027, Japan
8. Graduate School of Pharmaceutical Sciences, Tohoku University, Sendai, Miyagi, 980-8578, Japan

+ These authors contributed equally.

* To whom correspondence should be addressed. Tel: +81 22 274 6040; Fax: +81 22 274 6040; Email: kengo@tohoku.ac.jp

```
query {
  metabolite(where: { commonName: { _eq: "Glycine" } }) {
    metaboliteId

    gwasTopHitSummaries {
      gwasStudy {
        gwasStudyId
        title
      }

      variationSummary {
        variationId
        chromosome
        position
        reference
        alternative

        tommoFreq
        gnomadFreq
      }

      pvalue
      beta
    }
  }
}
```

Searches metabolite by name "Glycine"

Retrieves top-hit genome variation and GWAS analysis information associated to a metabolite

Retrieves the GWAS study description

Retrieves information on top-hit genomic variation (Retrieves variation ID, chromosome, position, alleles, and allele frequencies from WGS panels),

Retrieves GWAS results (p-value and beta value)

Supplementary Figure S1: An example of GraphQL query for the jMorp database. This GraphQL query retrieves metabolites with the name "Glycine," GWAS analysis results (top-hit genome variation, p-value, beta) associated with the metabolite.

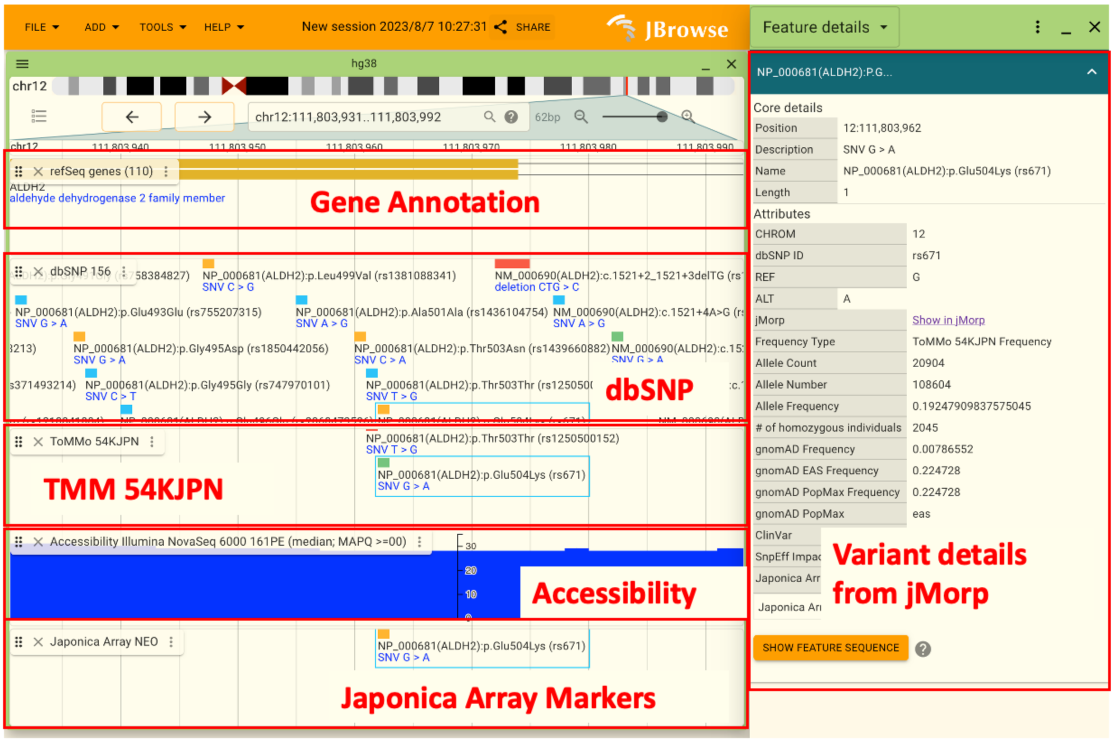

Supplementary Figure S2: Genome browser example. In this figure, tracks for displaying genome-related data, including genome variation information (TMM 54KJPN track), maker sites of Japanese-specific SNV array (Japonica Array Markers track), and genome accessibility data (Accessibility track), as well as publicly available datasets, such as gene annotation and dbSNP are shown.

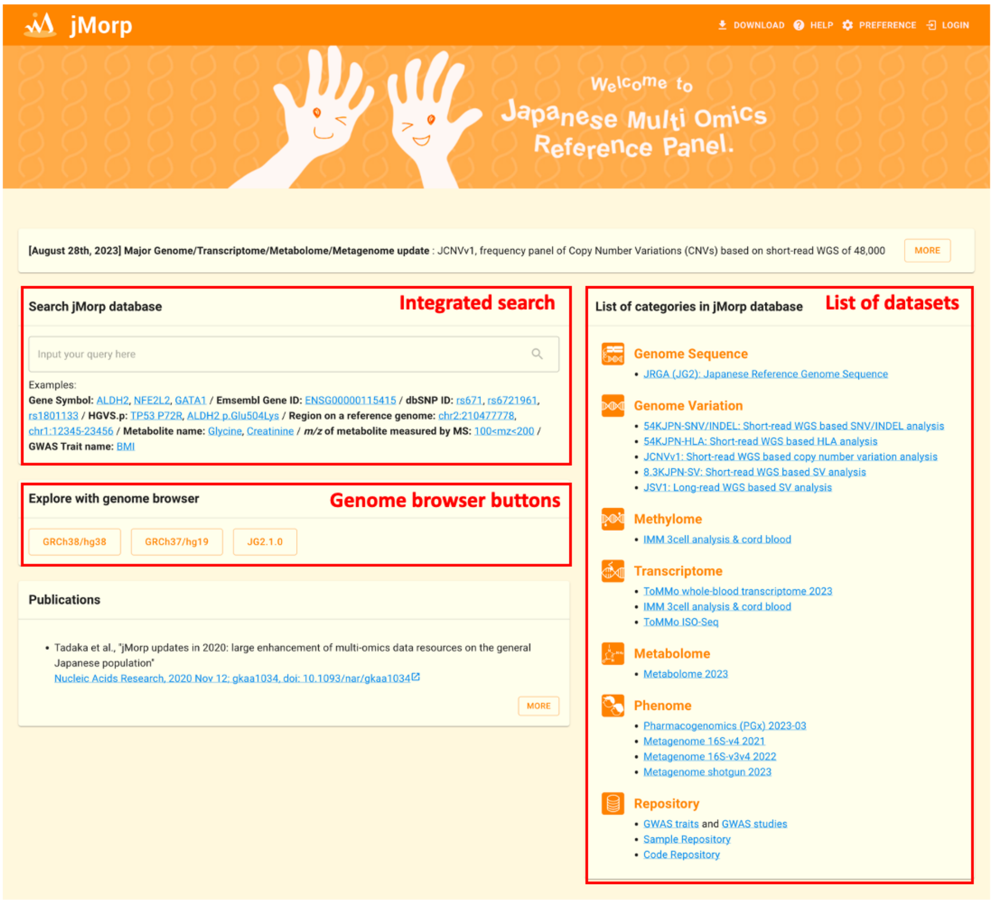

Supplementary Figure S3: Top page of the jMorp website. A search box is in the upper left corner of the top page, and it can be used to perform integrated searches on all data contained in jMorp. The search box accepts various search terms, including gene symbols, gene IDs, dbSNP IDs, HGVS.p, coordinates on the genome, metabolite names, m/z of metabolites measured by MS, and GWAS trait names. In the lower-left corner of the top page, three buttons to launch genome browsers are located. Genome browsers allow users to check genome-related data such as SNV/INDEL site information, methylated site information, and GWAS Manhattan plots in one centralized view. A list of datasets included in jMorp is displayed on the right side of the top page. Users can see a list of entries in that dataset by clicking on the name of a dataset. Summaries of each dataset are available in supplementary table S1.

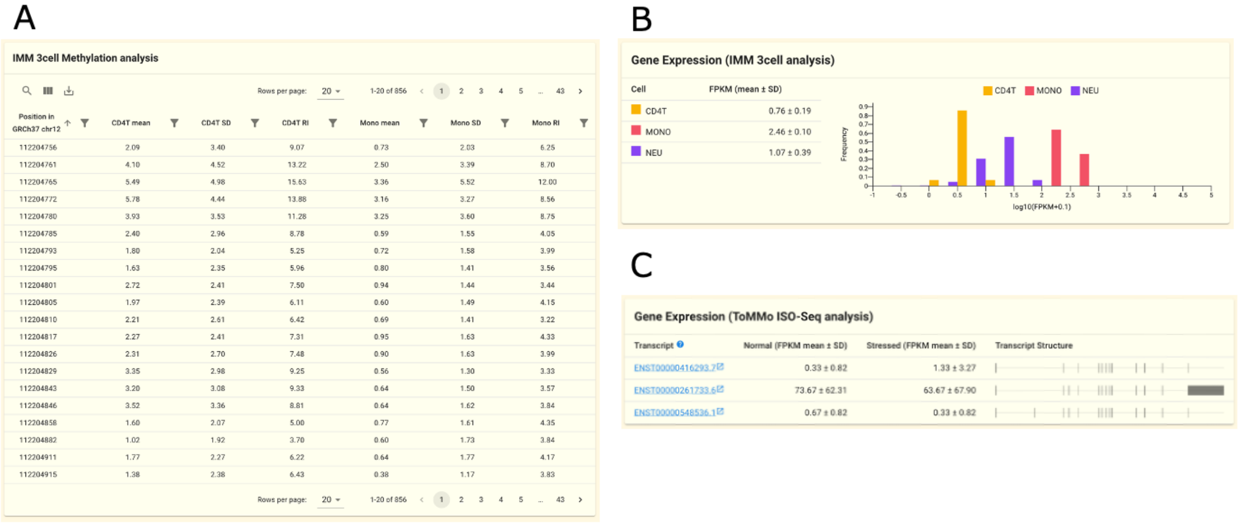

Supplementary Figure S4: Panels shown in the gene page but omitted from Figure 2 due to figure size limit. (A) Methylation analysis result panel shown on the gene page for the ALDH2 gene. The coordinates on the GRCh37 reference genome sequence and the mean, variance, and reference interval of the methylation level at that position are displayed for each cell type. (B) Transcriptome analysis result panel shown on the gene page for the ALDH2 gene. Mean and SD of the expression levels are shown for each cell type. (C) ISO-Seq analysis result panel shown on the gene page for the ALDH2 gene. One row of the table corresponds to one transcript, and the FPKM value (mean and standard deviation) for each transcript is displayed. Two types of FPKM values are displayed: normal and under stress. In addition, a simple drawing of the structure of each transcript is displayed on the right side of the table. In the figure, squares correspond to exons.

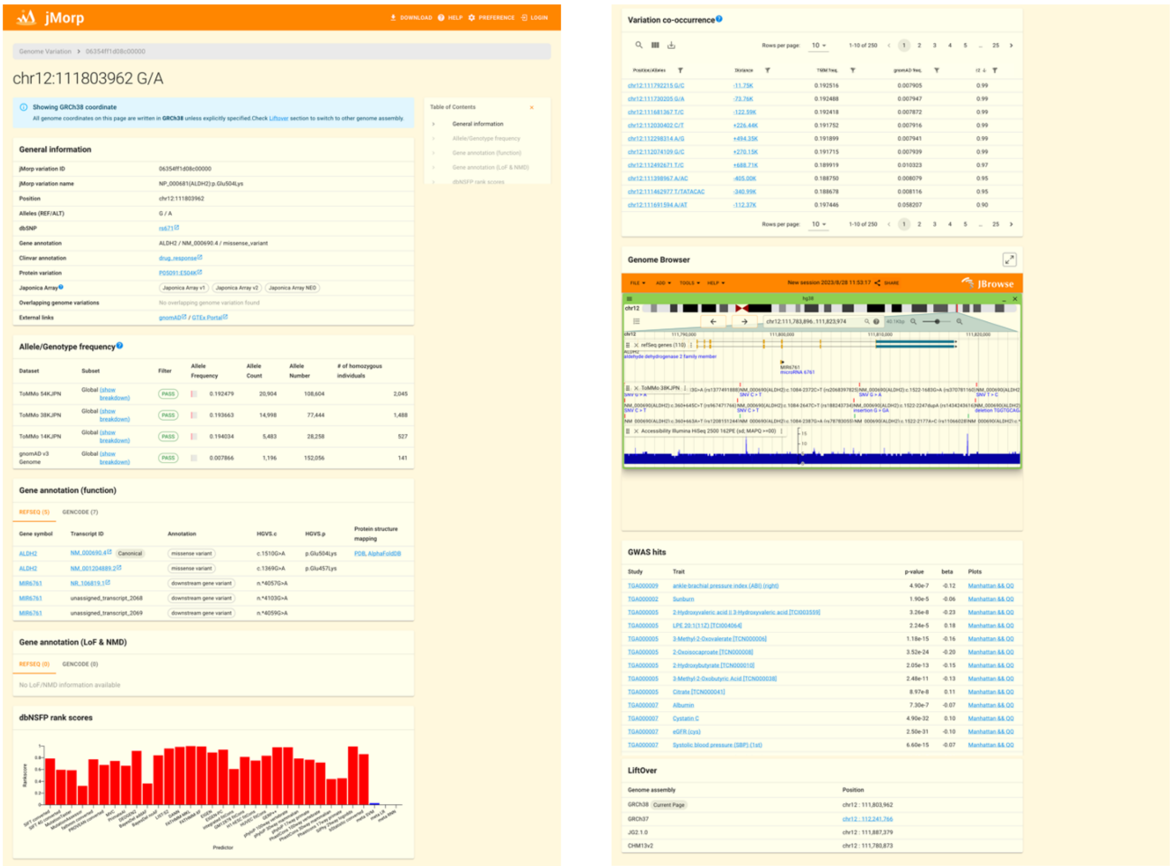

Supplementary Figure S5: SNV/INDEL page for the rs671 genome variation. SNV/INDEL page collects detailed information about a genome variation, including functional annotations (HGVS.c, HGVS.p) and ClinVar annotations, dbSNP IDs, allele frequency information in the TMM whole genome panel, and gnomAD database, and severity scores in dbNSFP, GWAS analysis results. In addition, users can move from the genome layer to other layers, such as the metabolome, by clicking on the trait name displayed in the GWAS analysis results panel.

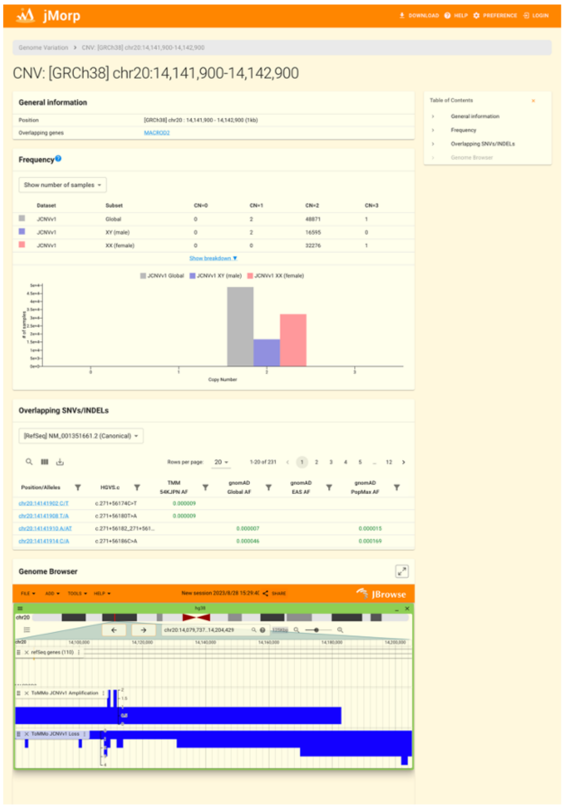

Supplementary Figure S6: CNV page for analysis result of chr:22:14,141,900-14,142,900 region. The “frequency” panel shows the number of samples by copy number. The “Overlapping SNVs/INDELs” panel displays a list of SNVs and INDELs whose locations are in chr:22:14,141,900-14,142,900. The Genome Browser panel is present at the bottom of the page, and users can examine the genome region near the CNV.

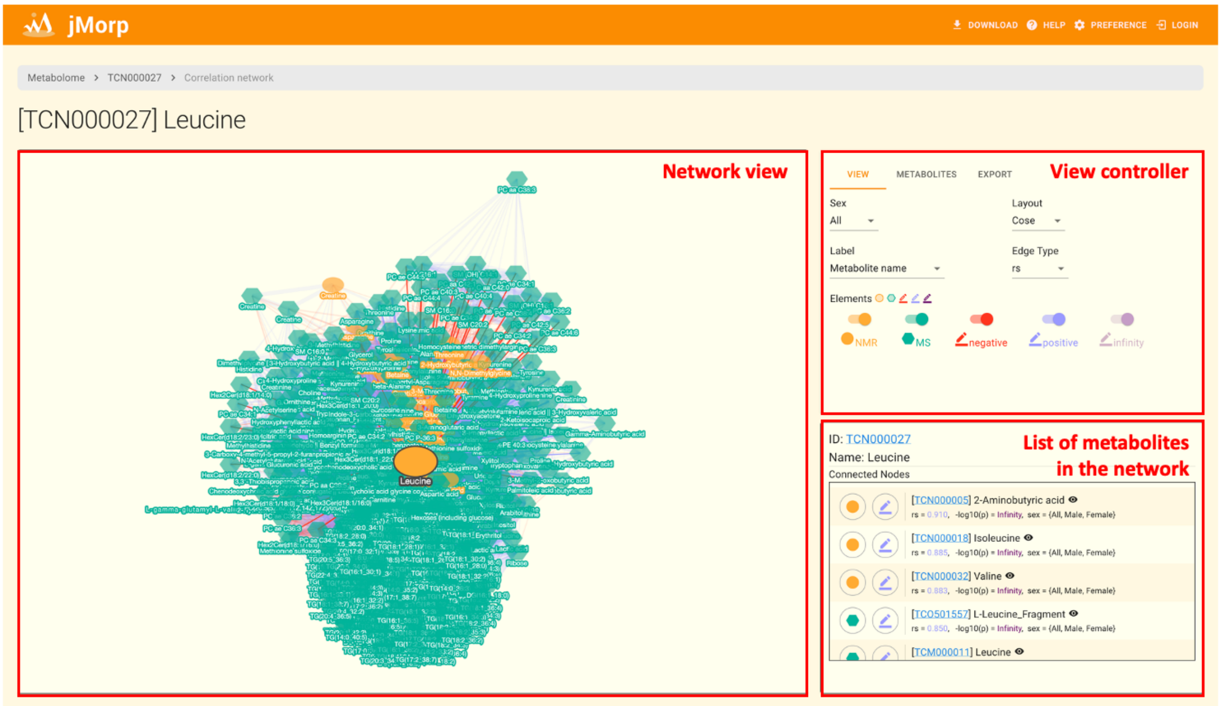

Supplementary Figure S7: Correlation network viewer for Leucine (TCN000027). This page mainly contains three blocks. At the left side of the page, a correlation graph is drawn. Nodes and edges on the graph correspond to metabolite and correlation between two metabolites, respectively. The color of a node indicates the measurement method of each metabolite. (NMR: orange, MS: green), The color and opacity of an edge represent the direction and strength of correlation. (red: negative correlation, blue: positive correlation). The "view controller" block is placed at the top-right of the page. Users can use this block to perform various operations on the network, such as displaying only negative correlations. A list of metabolites in the network is displayed at the bottom right of the page.

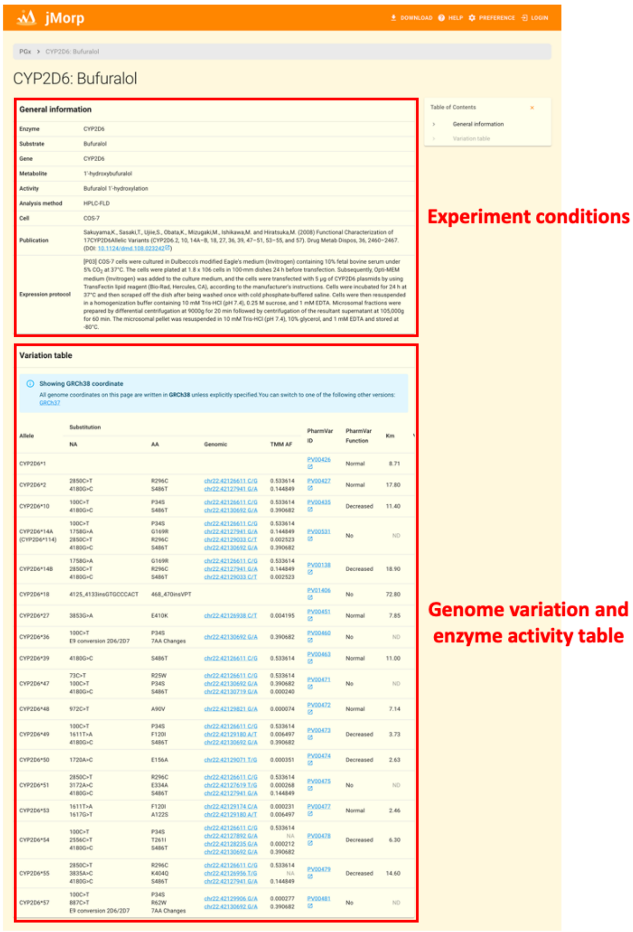

Supplementary Figure S8: An example of a PGx page. The enzyme activity measurement experiment conditions and references are displayed at the top of the page. At the bottom of the page, a table showing the relationship between genome variations and enzyme activity measurement results is displayed. Each genomic variant in the table has a link to the SNV/INDEL page, allowing the user to check the detailed information of the genomic variant quickly.

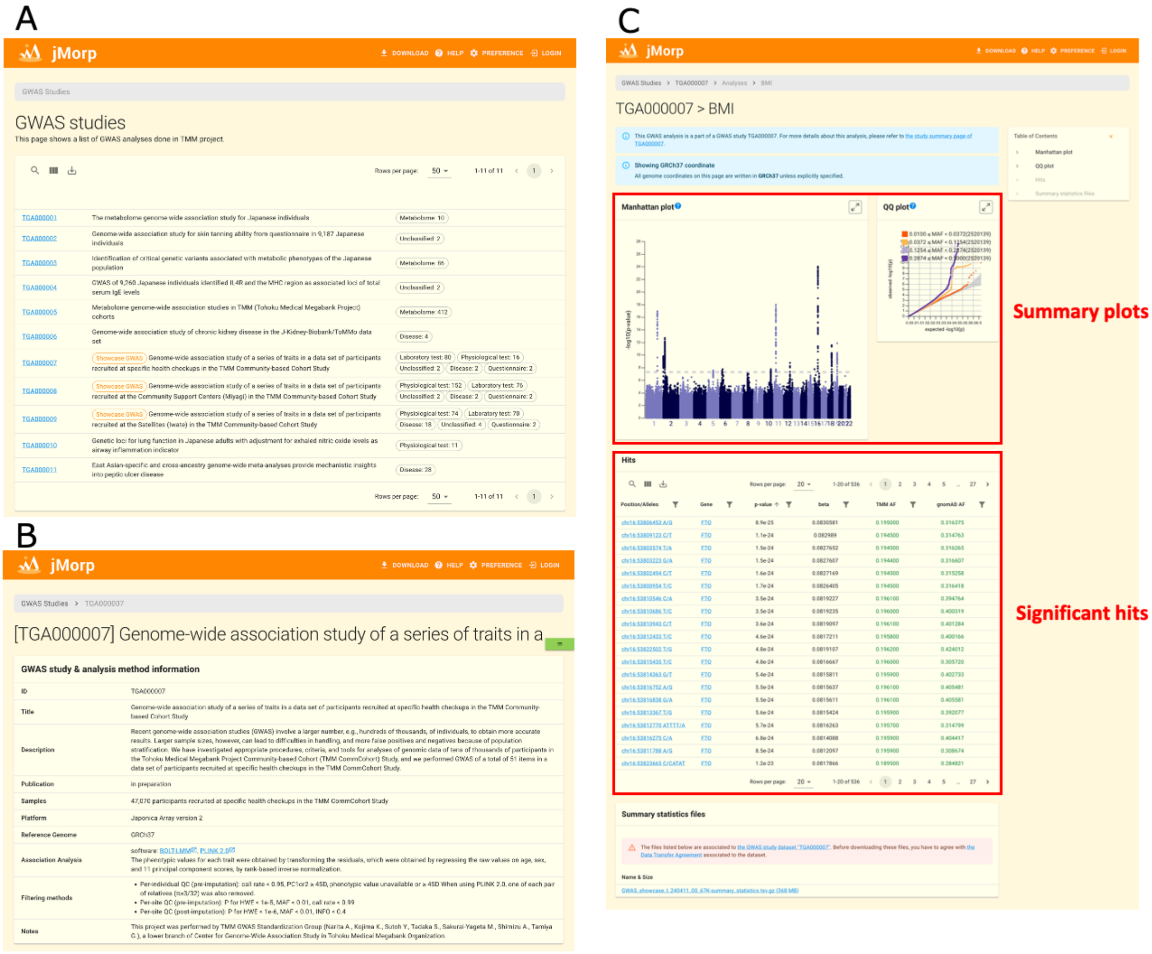

Supplementary Figure S9: Examples of GWAS pages. (A) Screenshot of a GWAS study list page. The current version of jMorp contains 11 studies. (B) Screenshot of a GWAS study detail page. This page is prepared for each GWAS study (publication) and contains the title and summary of the survey and GWAS method summary. (C) Screenshot of a GWAS analysis detail page. In the figure, the page displays the results of GWAS analysis with BMI as the phenotype. The Manhattan plot and QQ plot are shown at the top of the page, and the table of significant hit variations is displayed at the bottom.

Supplementary Table S1: Samples included in the 54KJPN panel.

(a) Number of samples according to age and sex.

Age	Male	Female	Total
**19 and below**	233	1,292	525
**20-29**	1,099	5,817	6,916
**30-39**	2,865	10,024	12,889
**40-49**	1,686	3,037	4,723
**50-59**	2,030	4,420	6,450
**60-69**	7,047	9,329	16,376
**70-79**	3,246	3,077	6,223
**80 and above**	57	43	100
**Total**	18,263	36,039	54,302

(b) Number of samples according to analysis platform and sex.

Analysis platform	Protocol	Male	Female	Total
**Illumina HiSeq 2500**	162PE	1,450	1,677	3,127
	259PE	142	189	331
**Illumina HiSeq X Five**	150PE	32	35	67
**Illumina NovaSeq 6000**	150PE	4,913	15,387	20,300
	151PE	1,605	2,301	3,906
	161PE	6,291	11,624	17,915
**MGI DNBSeq G400**	150PE	620	671	1,291
**MGI DNBSeq T7**	150PE	3,210	4,155	7,365
**Total**		18,263	36,039	54,302

Supplementary Table S2: Samples included in the JCNVv1 panel.

(a) Number of samples by age and sex.

Age	Male	Female	Total
**19 and below**	220	275	495
**20-29**	1,013	5,234	6,247
**30-39**	2,607	8,895	11,502
**40-49**	1,528	2,685	4,213
**50-59**	1,852	3,960	5,812
**60-69**	6,399	8,446	14,845
**70-79**	2,932	2,746	5,678
**80 and above**	46	36	82
**Total**	16597	32277	48874

(b) Number of samples by analysis platform and sex.

Analysis platform	Protocol	Male	Female	Total
**Illumina HiSeq 2500**	162PE	1,347	1,573	2,920
	259PE	134	180	314
**Illumina HiSeq X Five**	150PE	32	34	66
**Illumina NovaSeq 6000**	150PE	4,418	13,371	17,789
	151PE	1,532	2,139	3,671
	161PE	5,765	10,682	16,447
**MGI DNBSeq G400**	150PE	574	611	1,185
**MGI DNBSeq T7**	150PE	2,795	3,687	6,482
**Total**		16,597	32,277	48,874

Supplementary Table S3: A summary of samples in the whole blood transcriptome dataset.

Age group	Sex	Number of samples
**30s**	**Female (pregnant)**	14
**30s**	**Female (non-pregnant)**	106
**60s**	**Female**	172
**30s**	**Male**	109
**60s**	**Male**	175
**Total**		576

Supplementary Table S4: Summary of metabolome analysis methods and number of samples analyzed.

Analysis method	Analysis Platform	Number of samples
NMR	NMR (Bruker: 600MHz)<br>CryoProbe SampleJet	63,577 samples<br>(Non-pregnant: 49,188, Pregnant: 14,389, Repeat survey: 4,597)
LC-MS/G-Met v1	C18 column: UHPLC-Q-TOF/MS (Waters: Synapt G2-Si)<br>HILIC column: HPLC-Q-FT/MS (Thermo Fisher Scientific: QExactive)	1,265 samples
LC-MS/G-Met v2	HPLC-Q-FT/MS (Thermo Fisher Scientific: QExactive)	2,971 samples
LC-MS/T-Met	UHPLC-MS/MS (Thermo Fisher Scientific: TSQ Quantiva)	2,363 samples
LC-MS/kit180	UHPLC-MS/MS (Waters: Xevo TQ-S)	1,482 samples (Repeat survey: 579)
LC-MS/kit500	UHPLC-MS/MS (Waters: Xevo TQ-XS)	9,247 samples (Repeat survey: 1,340)
GCMS/T-Met	GC-MS/MS (Shimadzu: TQ8040)	4,608 samples (Repeat survey: 645)

Supplementary Table S5: Datasets included in the jMorp database, their locations on the jMorp website and difference from jMorp 2020.

Category	Name	Content	Related pages on the jMorp website	Difference from jMorp 2020
**Genome (sequence)**	JG 2.1.0	Japanese reference sequence	Genome browser	(unchanged)
**Genome (variation)**	54KJPN – SNV/INDEL	Allele frequency information of SNVs and short INDELs based on short-read WGS of approximately 54,0000 individuals	SNV/INDEL page, Gene page	Number of samples analyzed increased from approxymately 8300 to 54,000
	54KJPN - HLA	HLA allele frequency information based on short-read WGS of approximately 54,000 individuals	Gene page	Newly added
	JCNVv1	Copy Numver Variation (CNV) frequency information based on short-read WGS of approximately 48,000 individuals	Gene page	Newly added
	8.3KJPN - SV	Structural Variation (SV) allele frequency information based on short-read WGS of approximately 8,300 individuals	Genome browser	Newly added
	JSV1	Structural Variation (SV) allele frequency information based on long-read WGS of 222 individuals	Genome browser	Newly added
**Genome (other)**	Genetic Map	Linkage disequilibrium maps calculated from 192 haploid genomes	Genome browser	Number of samples analyzed increased from 96 to 150
	SNV/INDEL co-occurrence	Co-occurrence (correlation) information of SNVs and INDELs calculated from 54KJPN-SNV/INDEL	SNV/INDEL page	Newly added
	Japonica Array Marker	Marker information on the SNP array "Japonica Array" constructed based on the TMM's Whole Genome Reference Panel 3.5KJPNv2, predecessor of 54KJPN	SNV/INDEL page, Genome browser	(unchanged)
**Methylation**	IMM 3-cell analysis	Short-read WGBS analysis of CD4T cells and monocytes isolated from 100 Japanese, powered by iMETHYL database	Gene page, Genome browser	(unchanged)
	IMM coordblood	WGBS analysis of coordblood of 100 Japanese, powered by iMETHYL database	Genome browser	Newly added
**Transcriptome**	IMM 3-cell analysis	Short-read transcriptome analysis of CD4T cells and monocytes isolated from 100 Japanese, powered by iMETHYL database	Gene page	(unchanged)
	ToMMo ISO-Seq	Long-read based transcriptome analysis of 3 Japanese individuals	Gene page	Newly added
	ToMMo whole blood	Short-read transcriptome of whole blood of 570 individuals	Gene page	Newly added
**Metabolome**	ToMMo metabolome 2023	Plasma metabolome analysis of 63,000 Japanese	Metabolite page	Number of samples analyzed increased from 23,000 to 63,000
**Phenome**	Pharmacogenomics (PGx)	Genome variations and enzyme activity in enzymes associated with drug sensitivity (15 genes, 382 genome variations (SNVs and INDELs)	PGx page	Newly added
	Metagenome 16S v4	Microbiome analysis data of plaque/saliva samples (16S v4 region analysis)	Metagenome page	Newly added
	Metagenome 16S v3/v4	Microbiome analysis data of plaque/saliva samples (16S v3/v4 region analysis)	Metagenome page	Newly added
	Metagenome shotgun	Microbiome analysis of feces samples of 315 Japanese	Metagenome page	Newly added
**GWAS**	GWAS summary statistics rep	Repository for GWAS summary statistics files to collect GWAS analyses performed in the TMM project	GWAS page, Genome browser	Number of GWAS traits increased from 2 to 401
